# Genome-wide association analysis identifies new candidate risk loci for familial intracranial aneurysm in the French-Canadian population

**DOI:** 10.1038/s41598-018-21603-7

**Published:** 2018-03-12

**Authors:** Sirui Zhou, Ziv Gan-Or, Amirthagowri Ambalavanan, Dongbing Lai, Pingxing Xie, Cynthia V. Bourassa, Stephanie Strong, Jay P. Ross, Alexandre Dionne-Laporte, Dan Spiegelman, Nicolas Dupré, Tatiana M Foroud, Lan Xiong, Patrick A. Dion, Guy A. Rouleau

**Affiliations:** 1 Montreal Neurological Institute and Hospital, McGill University, Montréal, QC Canada; 2 Department of Medicine, Faculty of Medicine, Université de Montréal, Montréal, QC Canada; 3 Department of Human Genetics, McGill University, Montréal, QC Canada; 4 Department of Medical and Molecular Genetics, Indiana University School of Medicine, Indianapolis, IN USA; 5 Faculty of Medicine, Université Laval, Quebec, QC Canada; 6 Centre de recherche, Institut universitaire en santé mentale de Montréal, Montréal, QC Canada; 7 Department of Neurology and Neurosurgery, McGill University, Montréal, QC Canada

## Abstract

Intracranial Aneurysm (IA) is a common disease with a worldwide prevalence of 1–3%. In the French-Canadian (FC) population, where there is an important founder effect, the incidence of IA is higher and is frequently seen in families. In this study, we genotyped a cohort of 257 mostly familial FC IA patients and 1,992 FC controls using the Illumina NeuroX SNP-chip. The most strongly associated loci were tested in 34 Inuit IA families and in 32 FC IA patients and 106 FC controls that had been exome sequenced (WES). After imputation, one locus at 3p14.2 (*FHIT*, rs1554600, p = 4.66 × 10^–9^) reached a genome-wide significant level of association and a subsequent validation in Nunavik Inuit cohort further confirmed the significance of the *FHIT* variant association (rs780365, FBAT-O, p = 0.002839). Additionally, among the other promising loci (p < 5 × 10^−6^), the one at 3q13.2 (rs78125721, p = 4.77 × 10^−7^), which encompasses *CCDC80*, also showed an increased mutation burden in the WES data (*CCDC80*, SKAT-O, p = 0.0005). In this study, we identified two new potential IA loci in the FC population: *FHIT*, which is significantly associated with hypertensive IA, and *CCDC80*, which has potential genetic and functional relevance to IA pathogenesis, providing evidence on the additional risk loci for familial IA. We also replicated the previous IA GWAS risk locus 18q11.2, and suggested a potential locus at 8p23.1 that warrants further study.

## Introduction

Intracranial Aneurysm (IA) has a prevalence of 1–3% in the general population^1,2^. The rupture of an IA can lead to subarachnoid hemorrhages (SAH), which has devastating consequences. Environmental and genetic factors, such as hypertension and smoking^3^, family history and ethnicity all contribute to the risk of IA. Because of the complexity of IA, genome-wide association studies (GWAS) have become the predominant strategy used to look for genetic factors associated with IA. These studies used several large cohorts with IA patients, mainly of Finnish, Japanese or European descent. Several risk loci were discovered in these GWA studies: 8q11.23 (*SOX17*), 9p21.3-23.1 (*CDKN2A-CDKN2BAS*)^4,5^ and 2q33.1 from the European and Japanese cohorts; 18q11.2 (*RBBP8*), 13q13.1 (*STARD13-KL*) and 10q24.32^6^ from the Finnish and Japanese cohorts; 1q23.1, 3p25.2, 7p21.2, 9q31.3^6^ and 4q31.22 (*EDNRA*)^7^ from two Japanese cohorts, and 7p21.1 (*HDAC9*)^8^ from a cohort with European ancestry. A more recent Finnish IA study revealed additional GWAS risk loci, including 2q23.3, 5q31.3 and 6q24.2, represented by low-frequency SNPs^9^. Multiple GWAS signals suggest that the genetic etiology of IA may be complex and population specific. The risk loci found in each GWA study was estimated to only account for 4.1–6.1% of the heritability in the respective cohort^9^.

It has been reported that the French-Canadian (FC) population has higher IA/SAH incidence and that patients usually aggregate in large pedigrees^10^, with 30% of IA patients having a family history (fIA)^11^. Similarly, to the Finnish people, French-Canadians are also descended from a relatively small founder population and have population specific variants due to the population bottleneck and genetic drift. Therefore, we hypothesized that population specific and median/low frequency variants may play an important part in the disease risk in FC fIA.

## Results

### GWAS discovery phase

After data QC and sample pruning, 621,983 SNPs with 173 FC IA cases and 1,772 FC controls remained in the analysis. The genome-wide threshold for significance after Bonferroni correction was set to 5 × 10^−8^. Marker-wise P values of Cochran–Armitage trend test were performed using PLINK 1.9^12^ for genotyped variants. Genomic inflation factor λ = 1.02 indicated that there was little inflation of excessive significant markers, as shown in quantile-quantile (QQ) plot **(**Supplementary Figure S1**)**.

The result of the initial trend test showed 3q13.2 as the most significant locus: rs2705520 (p = 6.93 × 10^−8^), an intronic SNP in *ATG3* (*autophagy 3*), followed by rs1877362 (p = 1.16 × 10^−7^) in *CCDC80* (*coiled-coil domain containing 80*) and rs1472107 (p = 1.18 × 10^−7^) in *SLC35A5* (*Solute Carrier Family 35 member A5*) (Supplementary Figure S2).

**Figure 1:**
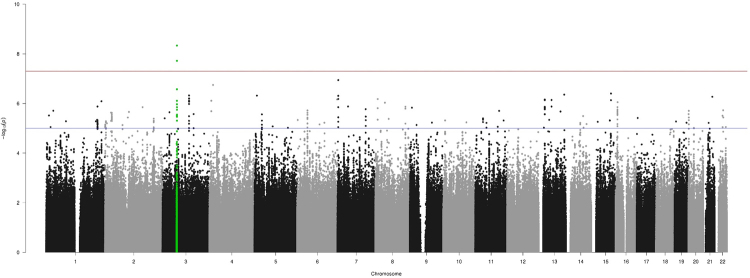
Genome-wide assocation analysis in the FC IA discovery cohort. Imputed using HRC panel, 7,614,484 variants passed QC are included in making the manhattan plot. X-axis shows the physical position along the genome. Y-axis shows the −log10 (p-value) for association. Red line indicates the level of genome-wide significant association (p = 5 × 10^−8^); blue line indicates the level of suggestive association (p = 5 × 10^−6^). Green dots indicate *FHIT* SNPs.

After imputation using the Haplotype Reference Consortium (HRC) and the exclusion of low quality and low MAF variants, 7,614,484 remaining variants were included in the test of associations using SNPtest^13^. The results were shown in the Manhattan plot (Figure 1). One locus reached genome-wide significant level after imputation: 3p14.2 (rs1554600, OR 0.26, p = 4.66 × 10^−9^) located in gene *FHIT*. TaqMan validation of rs1554600 on IA cases and controls suggested the imputation was accurate (maf.case = 0.0838 and maf.control = 0.026). The 28 most significant SNPs, representing 26 distinct loci that each reached suggestive level (p < 5 × 10^−6^), were prioritized for further validation (Table 1). A meta-analysis of 138 SNPs (Supplementary Table S1) located in 23 out of 26 loci comprising the current study and FIA cohort^8^ showed that three SNPs: rs76308736 (8p23.1), rs7084131 (10p14) and rs4867356 (5p13.3) are validated with decreased p-value (Table 2).

**Table 1:** 26 loci (28 genes) with top SNPs reached promising level of association.

loci	top SNP	Position (hg19)	Gene (or nearby)	Frequency in cases	Frequency in controls	OR	P-value	SE	Beta	INFO
3p14.2	rs1554600	61157774	*FHIT*	0.080925	0.022291	0.258936	4.66E-09	0.338309	−1.98215	0.94
7p22.2	rs12535623	2951412	*CARD11*	0.260116	0.154345	0.519156	1.15E-07	0.157048	−0.83267	0.95
4p15.32	rs116130729	16012786	*PROM1*	0.072254	0.023138	0.304125	1.80E-07	0.343542	−1.79302	0.86
3q13.2	rs78125721	112325677	*CCDC80*	0.043353	0.009029	0.201063	4.77E-07	0.492736	−2.4811	0.97
21q22.13	rs111610752	38151254	*HLCS*	0.052023	0.016366	0.303181	5.39E-07	0.430726	−2.15882	0.68
8p23.1	rs117537300	10743939	*XKR6*	0.118497	0.052765	0.414387	6.64E-07	0.244413	−1.21512	0.88
13q12.13	rs7989887	25681112	*PABPC3*	0.16185	0.087472	0.4964	6.93E-07	0.203382	−1.00943	0.99
13q14.3	rs114375292	54413853	*LINC00558*	0.080925	0.030756	0.360387	7.25E-07	0.313554	−1.55348	0.89
1q42.2	rs150148362	234191488	*SLC35F3*	0.040462	0.008183	0.195651	8.16E-07	0.517978	−2.55442	0.74
16p13.2	rs138031402	8960325	*CARHSP1*	0.043353	0.01044	0.232811	8.87E-07	0.489044	−2.40373	0.77
7p13	rs146929064	44346336	*CAMK2B*	0.060694	0.020598	0.325486	1.32E-06	0.38035	−1.83941	0.85
1q41	rs12058987	217421015	*ESRRG*	0.083815	0.035553	0.402958	1.33E-06	0.305576	−1.47758	0.98
8q24.21	rs7011138	127922200	*PCAT1*	0.092486	0.19526	2.38087	1.35E-06	0.149667	0.72319	0.99
9p23	rs2039332	9138642	*PTPRD*	0.049133	0.011005	0.215339	1.48E-06	0.42766	−2.05874	0.79
16p13.2	rs982855	8297685	*RBFOX1/TMEM114*	0.572254	0.437641	0.581703	1.78E-06	0.116681	−0.55742	0.99
6p21.1	rs6911069	43617687	*RSPH9*	0.069364	0.025113	0.34561	1.92E-06	0.346004	−1.6476	0.99
20p13	rs56040592	1611918	*SIRPG*	0.104046	0.051072	0.463459	1.98E-06	0.257248	−1.22327	0.98
3p24.1	rs142836448	29398714	*RBMS3*	0.063584	0.153217	2.66475	2.26E-06	0.166286	0.786315	0.95
2p23.3	rs77639126	25598423	*DTNB*	0.072254	0.023984	0.315525	2.35E-06	0.335271	−1.58283	0.91
2p23.2	rs13028204	28853137	*PLB1*	0.086705	0.041479	0.455814	2.39E-06	0.29275	−1.38092	0.98
3q22.1	rs114999403	131692910	*CPNE4*	0.049133	0.014955	0.293814	2.67E-06	0.428374	−2.01095	0.97
20p13	rs77402555	876841	*ANGPT4*	0.046243	0.012415	0.259286	2.69E-06	0.456509	−2.14253	0.89
5p13.3	rs72753560	31626036	*PDZD2*	0.086705	0.178612	2.29048	2.75E-06	0.14898	0.698537	0.97
22q12.3	rs150551568	33136068	*SYN3*	0.046243	0.011851	0.247359	3.12E-06	0.444976	−2.07483	0.95
14q24.2	rs117272176	72902205	*RGS6*	0.046243	0.014108	0.295149	3.23E-06	0.449474	−2.09269	0.77
2p33.3	rs1207426	206154365	*PARD3B*	0.16763	0.096501	0.530358	4.10E-06	0.189958	−0.87503	0.99
15q25.1	rs8032417	78569930	*DNAJA4*	0.393064	0.275959	0.588522	4.82E-06	0.129826	−0.59364	1
10p14	rs7084131	8399121	*GATA3*	0.011561	0.068002	6.23842	4.83E-06	0.226803	1.03693	0.99

**Table 2:** Three loci with SNPs validated in the FIA cohort.

LOCI	Marker	CHR	POS	Allele1	Allele2	Zscore	P-value	Direction	P.FC	SampleSize.FC	P.FIA	SampleSize.FIA
8p23.1	rs76308736	8	10572792	a	g	−4.753	2.01E-06	—	4.5E-06	1945	0.0093	4033
10p14	rs7084131	10	8399121	a	g	4.686	2.78E-06	++	4.83E-06	1945	0.0114	4033
5p13.3	rs4867356	5	31623146	t	c	−4.608	4.07E-06	—	4.91E-06	1945	0.0148	4033

Using imputed SNPs, the two loci 3p14.2 and 3q13.2 were estimated by GCTA-GREML^14–16^ (Genome-wide Complex Trait Analysis) to account for approximately 3% of the heritability in the FC cohort (standard error (SE) = 0.026).

### Replication in exome data and Inuit cohort

We looked into the exome sequencing data of the aforementioned 28 genes containing the top GWAS SNPs in our FC WES cohort; of the 23 genes that had exonic variants, a total of 177 exonic and splicing variants were found in 138 FC cases and controls. Sequence Kernel Association Test (SKAT)^17^ results showed excessive exonic variation burden in IA cases in four genes *SLC35F3* (p = 0.002), *DTNB* (p = 0.003), *CCDC80* (p = 0.0005) and *PABPC3* (p = 0.0001) (Table 3). However, the first two genes were less convincing with the limited number of variants in the testing (two variants for each). *PABPC3* was unlikely to be a risk gene for IA due to its human testis-specific expression. Thresholds Test (VT)^18^ focused on the selected genes revealed that only *CCDC80* (p = 0.01) in 3q13.2 reached the statistical significance after accounting for multiple testing.

**Table 3:** Exonic and splicing variants of 23 GWAS suggestive genes in 138 FC cases and controls from WES.

Gene	Loci	Number of tested variants	*P*-value (SKAT)
*ESRRG*	1q41	2	0.7073712
*SLC35F3*	1q42.2	1	0.002189217
*DTNB*	2p23.3	2	0.002747022
*PLB1*	2p23.2	20	0.1122009
*PARD3B*	2p33.3	13	0.9205563
*RBMS3*	3p24.1	1	0.354907
***CCDC80***	**3q13**.**2**	**8**	**0**.**0005070405**
*CPNE4*	3q22.1	1	0.7073712
*PROM1*	4p15.32	12	0.05065522
*PDZD2*	5p13.3	19	0.7212919
*RSPH9*	6p21.1	2	0.8510591
*CARD11*	7p22.2	4	0.8172528
*CAMK2B*	7p13	3	0.09754801
*PTPRD*	9p23	7	0.2814774
***PABPC3***	**13q12**.**13**	**54**	**0**.**0001097577**
*RGS6*	14q24.2	1	0.7073712
*DNAJA4*	15q25.1	2	0.5637608
*RBFOX1*	16p13.2	5	1
*CARHSP1*	16p13.2	2	0.3250554
*ANGPT4*	20p13	4	0.09886071
*SIRPG*	20p13	5	0.7073707
*HLCS*	21q22.13	6	0.6115834
*SYN3*	22q12.3	3	0.5083075

In the Nunavik Inuit IA cohort, after performing Family Based Association Test (FBAT)^19^ of the 2,429 SNPs within the 28 aforementioned genes and neighboring regions, 50 SNPs located in the *FHIT* gene region were with p < 0.05 (Supplementary Table S2), the most significant one being rs780365 (p = 0.002839). Although the associations were no longer significant after corrections of multiple testing (p < 0.00014, 2,429 variants in 353 independent tests), it could be due to the limited sample size, which still suggested that *FHIT* variants may likely still be associated with IA.

### Replication of previous GWAS risk loci

**Figure 2:**
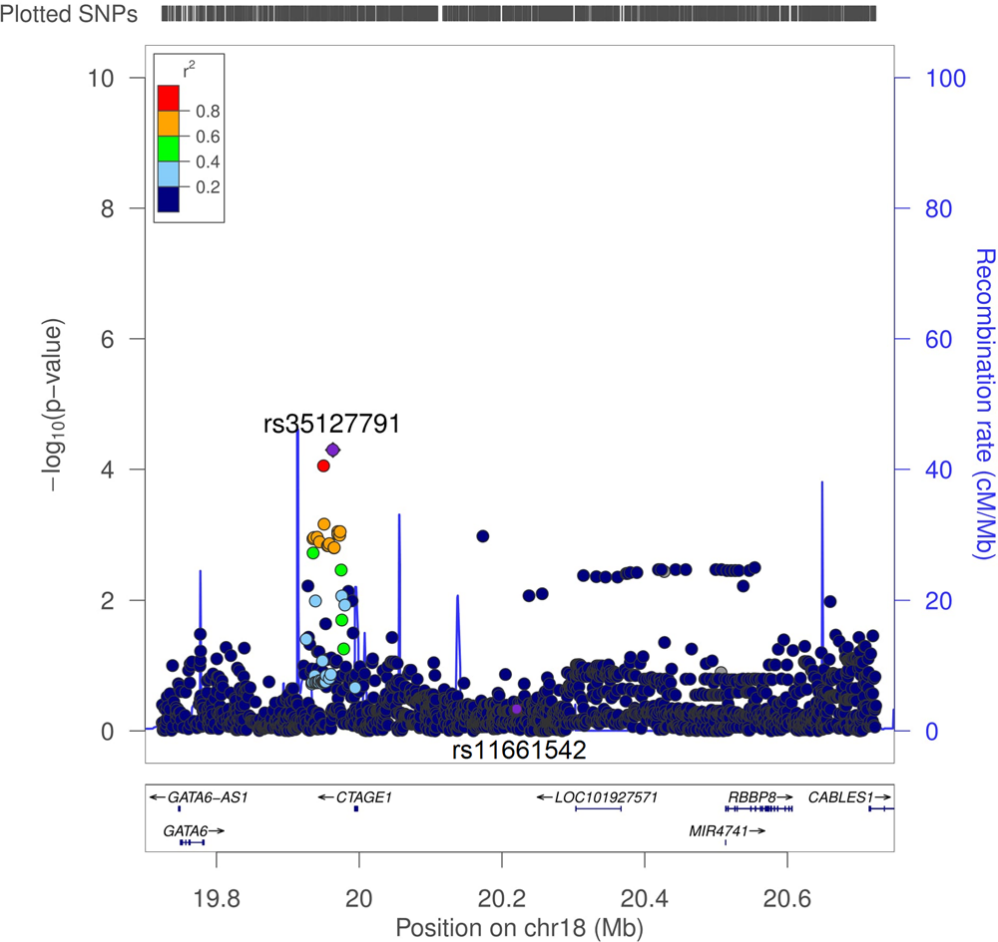
Regional association signals of the previous GWAS risk locus 18q11.2. LocusZoom plot showing the regional association of chr18.19.7-20.75 mb, including the most significant SNP rs35127791 and previous GWAS SNP rs11661542. Purple line indicates the genetic recombination rate (cM/Mb). SNPs in linkage disequilibrium with rs35127791 are shown in color gradient indicating r2 levels (hg19, 1KGP, Nov 2014, EUR).

We also attempted to replicate the 12 IA risk loci identified in previous GWAS in our FC IA cohorts. 825 distinct LD blocks were established in these 12 loci in the FC population, the level of significance was therefore p < 6.06 × 10^−5^ after multiple correction. As a result, only one SNP rs35127791 located in 18q11.2 was replicated (maf = 0.157, p = 5.05 × 10^−5^, beta = −0.66, SE = 0.16) (Figure 2). LocusZoom plots covering the rest of the 23 genome-wide significant SNPs from previous GWAS in these 12 loci were shown in Supplementary Figure S3.

We further looked into the FC WES data in locus 18q11.2, which comprised 4 protein coding genes: *GATA6*, *CTAGE1*, *RBBP8* and *CABLES1*, a variant burden test revealed exonic variants of *CABLES1* seemed to be associated with IA in FC (p = 0.022, corrected) (Supplementary Table S3).

## Discussion

**Figure 3:**
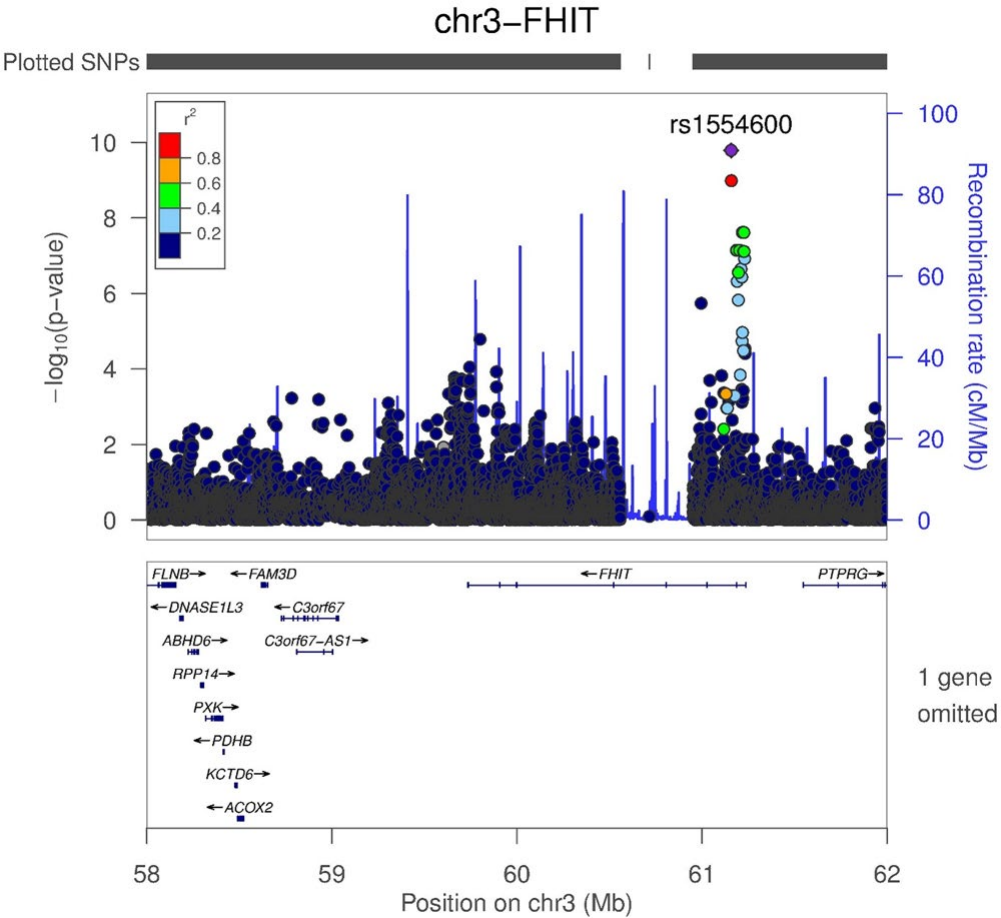
Regional association signals of 3p14.2 locus. 4 Mb region around the most significant association, rs1554600 in 3p14.2 locus is displayed using imputed data. Variant with the most significant association (rs1554600) is indicated in purple diamond. Purple line indicates the genetic recombination rate (cM/Mb). SNPs in linkage disequilibrium with rs1554600 are shown in color gradient indicating r2 levels (hg19, 1KGP, Nov 2014, EUR).

In this study, we discovered a new IA associated region on 3p14.2 which encompasses the *FHIT* gene in the French-Canadians (Figure 3), intronic variants in *FHIT* also suggested an association with IA in the Nunavik Inuit population. Additionally, we found evidence suggesting exonic variants in *CCDC80* within the 3q13.2 locus to be associated with the French-Canadian IA. Collectively, SNPs in *FHIT* and *CCDC80* could explain approximately 3% of the heritability of IA in French-Canadians, higher than that was reported in the Finnish study (2.1%)^9^ and in the GWAS replication (2.5%)^6^. The underlying reason for this might be that French-Canadians are a more homogenous population and this study has mainly included familial cases. On the other hand, we also replicated a previous GWAS risk locus 18q11.2^20^, the top SNP rs35127791 located approximately 200 kb upstream of *GATA6*, close to a H3K24Ac region in HUVEC cells. *GATA6* regulates the differentiative state of vascular smooth muscle cells, and an important candidate for cardiovascular development. *CABLES1* proves to have an important role in cancer and development of neurons^21^, a recent study also highlighted its function vascular cell senescence and inflammation through p21 regulation^22^.

Unfortunately, we could not replicate the most significant SNP in *FHIT* in another IA cohort. However, this was expected, as *FHIT* variants have higher MAFs in French-Canadians compared to other European populations. The signal may only exist in French-Canadian populations and may correlate with IA cases with hypertension. Among the three SNPs which were validated in the FIA cohort, rs76308736 is located in the promoter region of *SOX7*, with its crucial function in angiogenesis and establishment of arteriovenous identity. rs76308736 only showed suggestive significance in our discovery cohort and did not pass multiple correction in the FIA data, the relationship of this SNP and risk for IA remains to be explored.

Although the number of cases in this study was limited, we tried improving the power by doing the following: 1) targeting IA patients with family history, 2) focusing on individuals only with the French-Canadian ethnicity, and 3) validating the findings in exome sequencing results and in another founder population. Because French-Canadians originated from a small founder population, we also included intermediate variants after the imputation. The top SNPs that we discovered in *FHIT* and *CCDC80* had rare or intermediate frequency (2-4%). The MAF of rs1554600 was higher in French-Canadians (3.3%) compared to Europeans (1.9%) and was significantly higher when compared with East Asians (0.1%), which suggest a bottleneck and/or drift may be the reason for the accumulation of low-frequency variants that potentially associated with the risk of IA in the French-Canadians.

*FHIT* (fragile histidine triad) is a tumor suppressor gene that regulates DNA replication and signals stress responses^23^, and encompasses the most active of the common human chromosomal fragile regions (FRA3B). Its expression has an important role in response to oxidative damage^24^. On the other hand, oxidative stress is known to be a key contributor to IA formation and rupture^25^. Interestingly, a study highlighted that the SNPs in *FHIT* have been associated with hypertensive traits in populations from Saguenay-Lac-St-Jean region, mainly French-Canadians^26^. As over 40% of our French-Canadian IA cases were also affected with hypertension (Table 4), we considered that rs1554600 in *FHIT* is possibly more likely to be a risk for hypertensive IA in French-Canadians. Further test of *FHIT* SNPs between IA patients with and without hypertension revealed several SNPs in LD with rs1554600 to be significantly associated with this trait (ie. rs73098963, p = 0.002611, GWAS p value = 2.42 × 10^−8^). *FHIT* has been reported in other GWAS studies to associate with hypertension: rs6782531, which located at approximately 160 kb upstream and in high LD of rs1554600 have also been reported to be significantly associated with blood pressure^27^.

**Table 4:** FC sample demographics and the clinical information of IA cases.

**Sample demographic of cases and controls**		
	**Cases**	**Controls**
**Number**	257	1192
**Mean age of recruit (SD)**	53.9 (11.2)	54.4 (16.8)
**% Male**	31.5%	60.6%
**Clinical information of cases (212)**		
	**With family history**	**Sporadic**
**With clinical information**	147	65
**Multiple lesions (%)**	55 (37.4)	19 (29.2)
**SAH (%)**	51 (34.7)	25 (38.5)
**Hypertension (%)**	60 (40.8)	31 (47.7)
**Drinking (%)**	33 (22.4)	26 (40.0)
**Smoking (%)**	89 (60.5)	50 (76.9)
**Hypercholesterolemia (%)**	29 (19.7)	24 (36.9)

The 3q13.2 locus is a gene-rich region with many functions in inflammatory responses. *BTLA* (B- and T-lymphocyte attenuator) is involved in inflammatory responses^28^ and homeostasis of the immune system^29^. A previous study showed *BTLA* expression to be up-regulated in organs after a hemorrhagic shock^30^. The 3q13.2 locus also comprises *ATG3*, which encodes a protein known to induce apoptosis^31^ and also to act as a regulator of oxidant and inflammatory balance that regulates endothelial cell stress response^32^. The top SNP rs2705520 in *ATG3* was also reported in a GWAS to be associated with asthma^33^, suggesting its role in inflammatory diseases.

The most interesting gene in the 3q13.2 locus is *CCDC80*, also known as *SSG1* (steroid-sensitive gene 1), which is a cGMP signalling effector and was reported to be widely express in vascular smooth muscle cells^34^. A previous study also showed that it has a role as a modulator of glucose and energy homeostasis^35^. Another study highlighted that the product of fibroblast growth factor (*FGF*) regulates the expression of *CCDC80*^36^, which is in turn also involved in cell adhesion during differentiation of fibroblast^37^. *CCDC80* was reported as a tumor suppressor as well^38^. These evidences suggest the critical function of *CCDC80* in vascular formation. *CCDC80* harbor a large number of rare variants in the French-Canadians (Supplementary Table S4); which also showed a significant difference in the variation burden in cases and controls. Both *ATG3* and *CCDC80* are dosage sensitive genes^39,40^, therefore the potential different expression levels of those genes may affect the risk of IA.

In conclusion, we have provided evidence for four new loci associated with IA in French-Canadian IA cohort recruited from Montréal and Québec city, which could explain 3% of the disease heritability. Based on the findings of this study and the functions of their encoded products, two genes (*FHIT* and *CCDC80*) are potentially relevant to IA with strong aggregation of familial IA cases with high blood pressure in the French-Canadian population. *FHIT* is more particularly associated with hypertensive IA cases and this may be the consequence of a bottleneck and/or drift that affected the French-Canadian founder population. *CCDC80* was shown to have a large number of rare variations in the French-Canadian cohort and with a significantly different variation burdens between IA cases and controls. Both the lack of association of SNPs in *FHIT* and *CCDC80* and the replication of the only 18q11.2 locus of the previous GWAS hits suggests a genetic heterogeneity in IA, and thus additional studies targeting other high-risk populations are needed. However, the limited number of cases available in our study calls for a validation study that will have access to a larger cohort from the same founder population, thus to increase the power of detection.

## Methods

### Discovery cohort

The discovery cohort included 257 French-Canadian IA patients, a majority of them were with family history, which were recruited in Montréal and Québec City, Canada. The diagnoses were confirmed either by magnetic resonance angiography (MRA), or by surgical confirmation (clipped or coiled). An additional 1,992 controls, mainly comprised of unrelated FC individuals without cerebrovascular diseases were also included in the analysis. Their demographic information is listed in Table 4. Written informed consent were obtained from all participants, and this manuscript contain no identifying information for any participant. This study was approved by Comité d’éthique de la recherche du Centre hospitalier de l′Université de Montréal and McGill University ethics, all methods were performed in accordance with the relevant guidelines and regulations of McGill University (REB NEU-14-051).

### Genotyping and quality control

All patients and controls were genotyped using the Illumina NeuroX SNP-chip, which contains 719,885 markers and is comprised of the backbone of Illumina HumanOmniExpress-v24 BeadChip. Raw data was processed by Illumina GenomeStudio software before the genotypes were generated. Both markers and samples were passed through a series of quality control (QC) steps. Samples were first removed if duplicated or if they had one of the following issues: 1) sex discrepancies; 2) exceeded a missing rate of 0.02; 3) ethnical admixture determined by PCA; or 4) with cryptic relateness determined by PLINK. Markers were removed if they meet one of the following criteria: 1) exceeding a missing rate of 0.02; 2) having a minor allele frequency (MAF) lower than 0.01; 3) deviating from Hardy Weinberg Equilibrium (p < 0.0001).

Principal Component Analysis (PCA) impelemented in the package EIGENSOFT 6.0^41^ was performed to assess the ethnicity of the samples. Three distinct populations CEU, CHB and YRI from 1000 Genome (1KGP) Phase III were used for clustering and CEU outliners were removed from further analysis. The remaining homogeneous population was also adjusted for the principal components in the subsequent tests for associations.

### Imputation

Imputation was done by the Sanger Imputation Server (https://imputation.sanger.ac.uk/) using Haplotype Reference Consortium r1.1^41^, and were pre-phased using SHAPEIT2^43^. Imputed variants were included in the further analyses only with MAF >0.01 and with imputation quality score >0.3.

### Association analysis

Frequentist additive association implemented in SNPtest^13^ was used to test for association of the imputed dataset, between FC IA cases and controls. Five major principal components were used as covariates for ancestry adjustment along with the sex of samples. Only autosomal SNPs were analyzed.

Regional association of suggestive loci were plotted using LocusZoom^44^ with LD data from 1KGP CEU population.

### Heritability estimation

We estimated the heritability from the original and imputed variants within the most promising loci, using methods of Estimation of Variance explained by SNPs (GREML)^14^ and GREML-LDMS^16^ programs implemented in the package of the Genome-wide Complex Trait Analysis (GCTA)^15^.

### Meta-analysis

SNP identified in the current study that reached suggestive level of association (p < 5 × 10^−6^) were compared with the previous FIA GWAS summary statistics from 2,617 IA cases and 1,416 controls^8^. METAL was used to conduct meta-analysis of the two GWAS results for these selected SNPs.

### FC and Inuit IA WES data

We examined the loci from the GWAS signals that have reached suggestive level of association in the exome sequencing results of 32 selected FC IA cases and 106 FC controls. Variable Thresholds Test (VT)^18^ implemented in Variant Tools (Vtools)^45^ and Sequence Kernel Association Test (SKAT)^17^ were performed to test the exonic variation burden in the genes located in the GWAS significant regions.

Thirty-four Nunavik Inuit (Québec, Canada) families comprised of 49 IA patients and 124 family controls were also used to follow up the regions of significance. The samples were genotyped on Illumina HumanOmniExpress-v24 Beadchip which contains 730,525 SNPs. We looked into all the suggestive loci of the FC IA discovery cohort, and performed family-based association analysis (fbat) implemented in FBAT package^19^ in the Inuit SNP-chip data to test the case-control association in related individuals.

### Replication of previous IA GWAS loci

12 loci from previous GWA studies (Supplementary Figure S3) with 23 SNPs that reached genome-wide significance were selected to examine if they could be replicated in our study. 500 kb upstream/downstream of the first and last genome-wide significant SNP located in each of the 12 loci were investigated in our GWAS data. Multiple correction was performed on the number of independent tests, which was defined by the number of LD blocks within these 12 regions. LocusZoom was used for data plotting. The validated loci will further be examined in FC WES data using Genome Analysis Toolkit (GATK)^46^ and VTools.

## Electronic supplementary material

Supplementary material
