# Supplementary material for: Genome-wide association analysis identifies new candidate risk loci for familial intracranial aneurysm in the French-Canadian population
Source: Sci Rep. 2018 Mar 12;8:4356. doi: 10.1038/s41598-018-21603-7 (PMC5847615; doi:10.1038/s41598-018-21603-7)
Supplement: Supplementary file 1 — Supplementary material [file 41598_2018_21603_MOESM1_ESM.pdf]

Genome-wide association analysis identifies new candidate risk loci for familial intracranial aneurysm in the French-Canadian population

Authors and affiliations:

Sirui Zhou[1,2], Ziv Gan-Or[1,3], Amirthagowri Ambalavanan[1,3], Dongbing Lai[4], Pingxing Xie[1], Cynthia V. Bourassa[1], Stephanie Strong[1,3], Jay P. Ross[1,3], Alexandre Dionne-Laporte[1], Dan Spiegelman[1], Nicolas Dupré[5], Tatiana M Foroud[4], Lan Xiong[2,6], Patrick A. Dion[1,7], Guy A. Rouleau[1,7*]

1) Montreal Neurological Institute and Hospital, McGill University, Montréal, QC, Canada; 2) Department of Medicine, Faculty of Medicine, Université de Montréal, Montréal, QC, Canada; 3) Department of Human Genetics, McGill University, Montréal, QC, Canada; 4) Department of Medical and Molecular Genetics, Indiana University School of Medicine, Indianapolis, IN, USA; 5) Faculty of Medicine, Université Laval, Quebec, QC, Canada; 6) Centre de recherche, Institut universitaire en santé mentale de Montréal, QC, Canada; 7) Department of Neurology and Neurosurgery, McGill University, Montréal, QC, Canada.

***Corresponding author:**

Guy A. Rouleau, Department of Neurology and Neurosurgery, McGill University, Montréal (QC), Canada, H3A 2B4. Tel: 514-398-2690. E-mail: guy.rouleau@mcgill.ca.

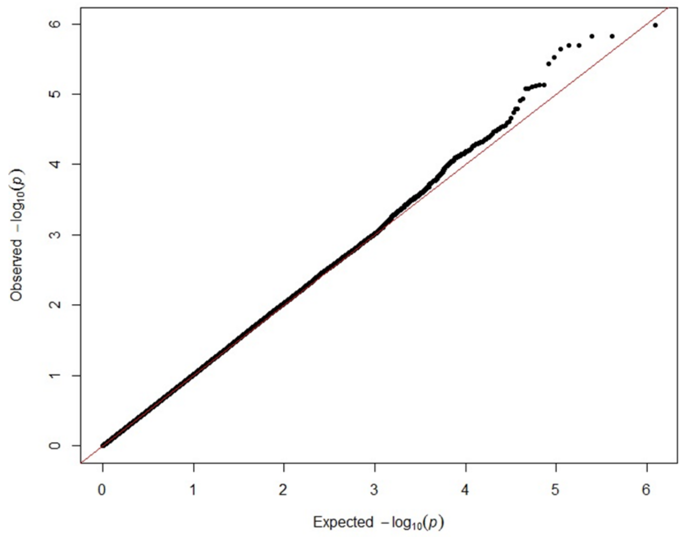

Supplementary Figure S1. Quantile-quantile plot of case-control logistic regression analysis

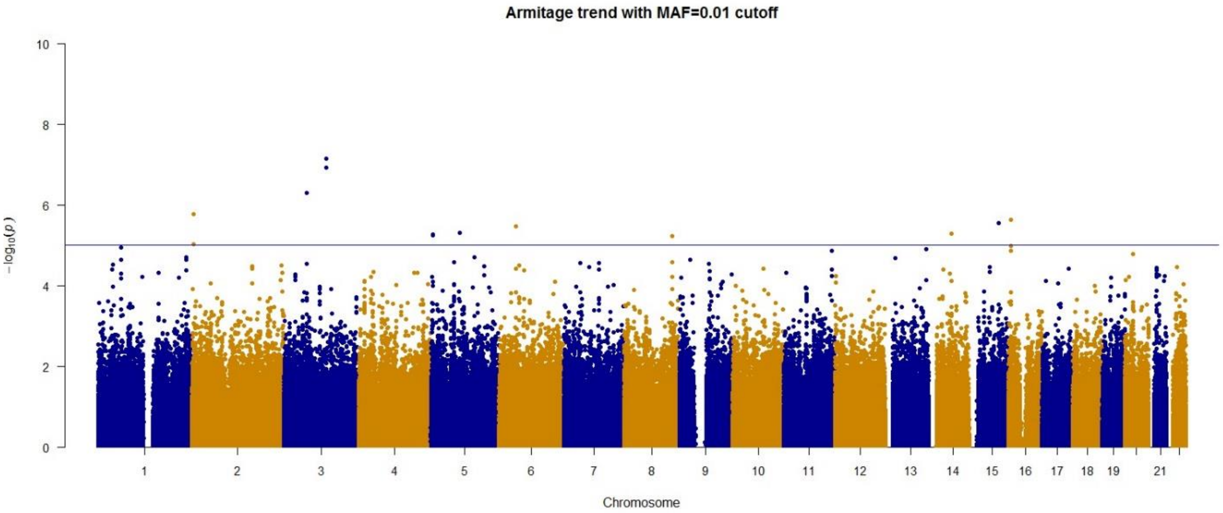

Supplementary Figure S2. Manhattan plot showing the result of the initial trend test

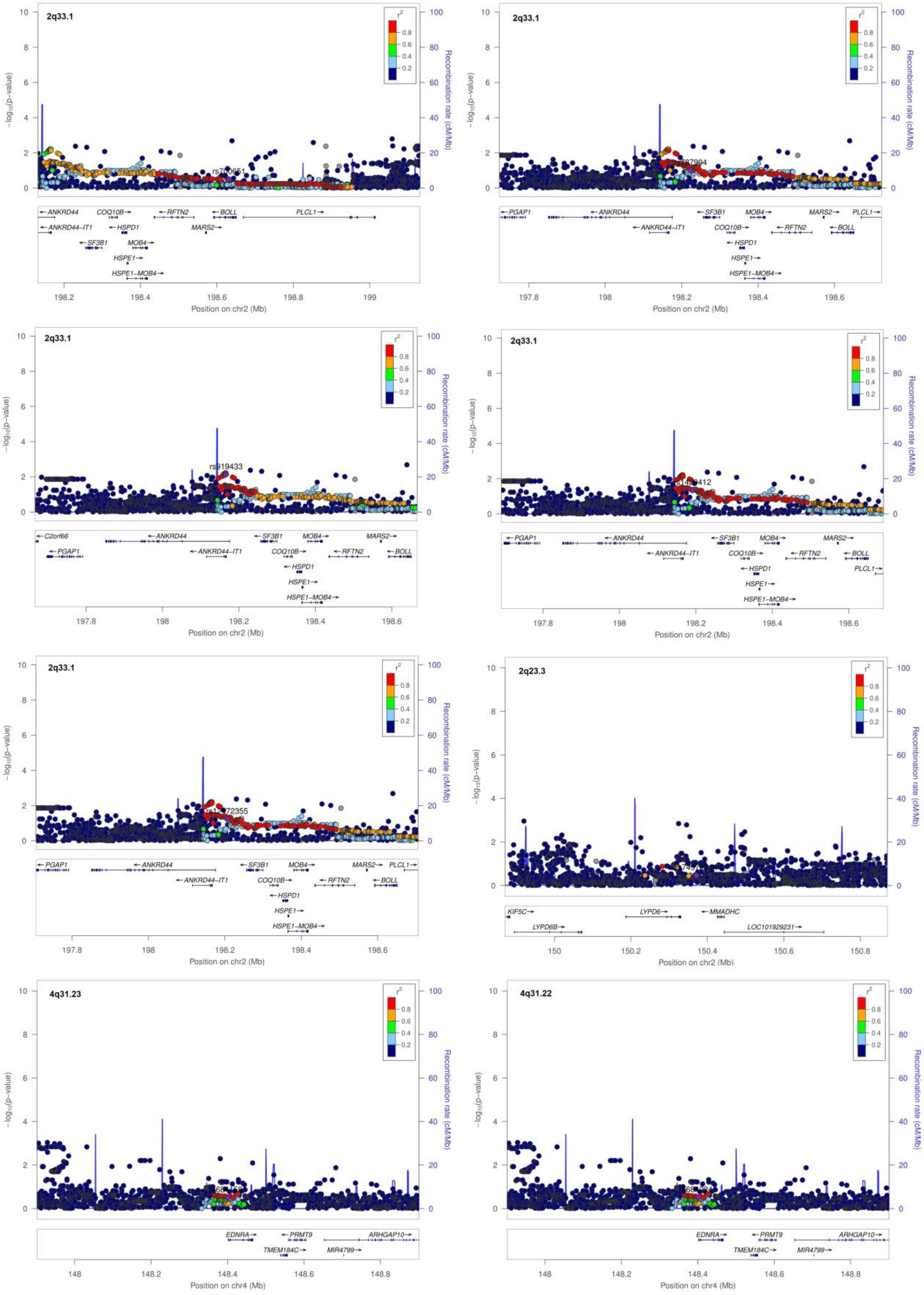
2q33.1
rs700651
ANKRD44
ANKRD44−IT1
SF3B1
COQ10B
HSPD1
MOB4
HSPE1
HSPE1−MOB4
RFTN2
BOLL
MARS2
PLCL1
Position on chr2 (Mb)
2q33.1
PGAP1
Position on chr2 (Mb)
2q33.1
rs919433
C2orf66
Position on chr2 (Mb)
2q33.1
Position on chr2 (Mb)
2q33.1
Position on chr2 (Mb)
2q23.3
KIF5C
LYPD6B
LYPD6
MMADHC
LOC101929231
Position on chr2 (Mb)
4q31.23
EDNRA
PRMT9
TMEM184C
ARHGAP10
MIR4799
Position on chr4 (Mb)
4q31.22
Position on chr4 (Mb)
−log10(p−value)
Recombination rate (cM/Mb)
r2

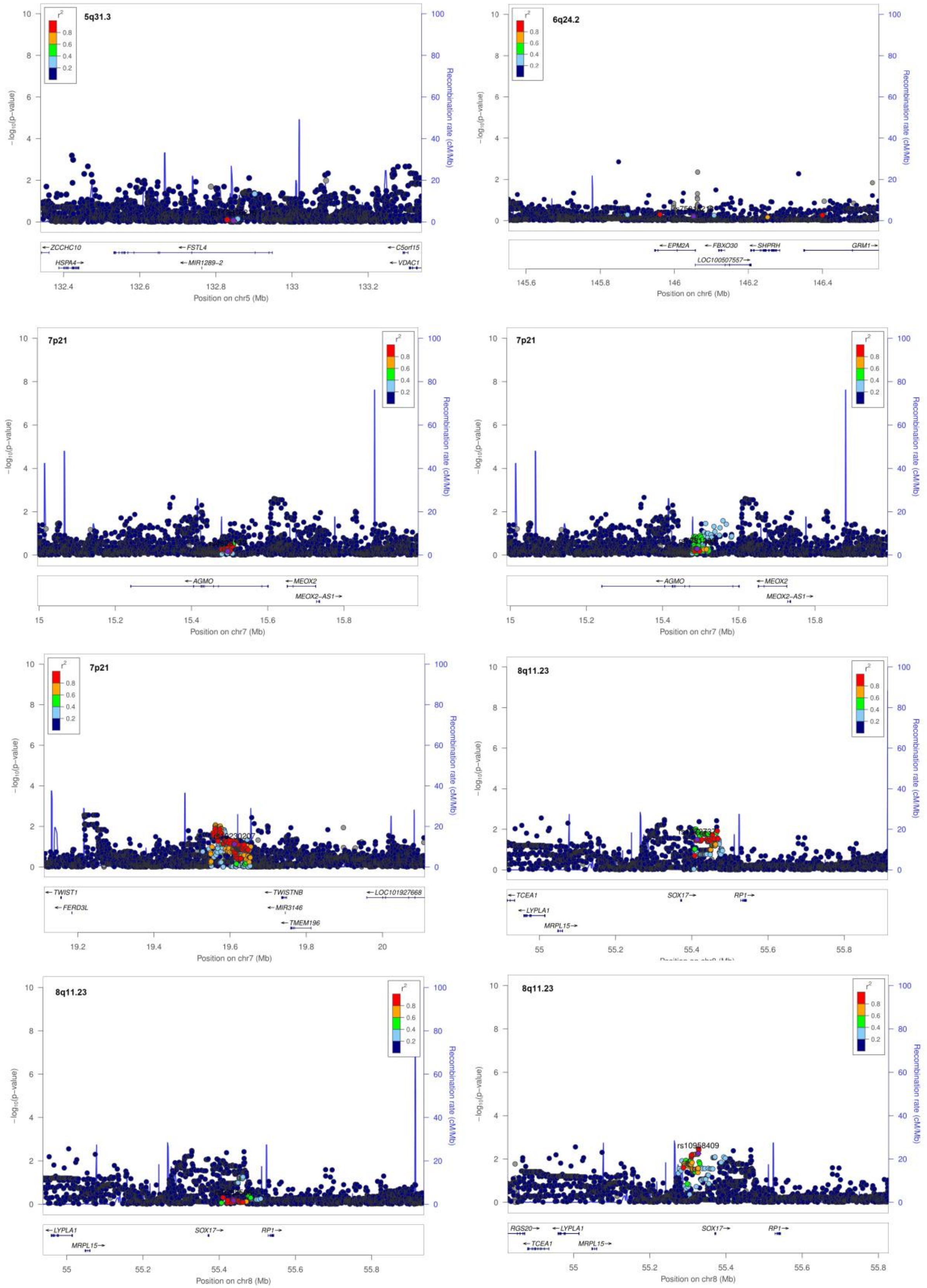
5q31.3
6q24.2
7p21
7p21
7p21
8q11.23
8q11.23
8q11.23
r2
0.8
0.6
0.4
0.2
−log10(p−value)
Recombination rate (cM/Mb)
ZCCHC10
FSTL4
C5orf15
HSPA4
MIR1289−2
VDAC1
Position on chr5 (Mb)
EPM2A
FBXO30
SHPRH
GRM1
LOC100507557
Position on chr6 (Mb)
AGMO
MEOX2
MEOX2−AS1
Position on chr7 (Mb)
TWIST1
FERD3L
TWISTNB
MIR3146
TMEM196
LOC101927668
TCEA1
LYPLA1
MRPL15
SOX17
RP1
RGS20
rs10958409
Position on chr8 (Mb)

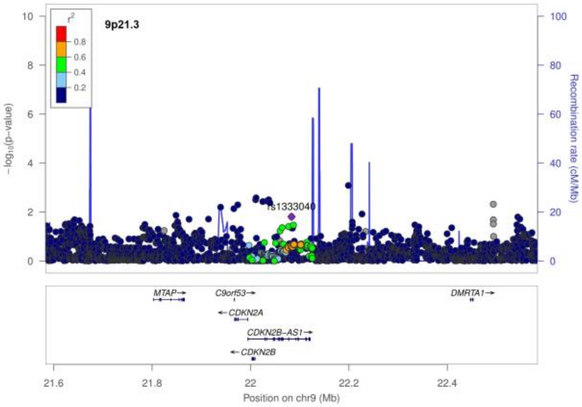
9p21.3
r2
rs1333040
−log10(p−value)
Recombination rate (cM/Mb)
MTAP→
C9orf53→
←CDKN2A
CDKN2B−AS1→
←CDKN2B
DMRTA1→
Position on chr9 (Mb)

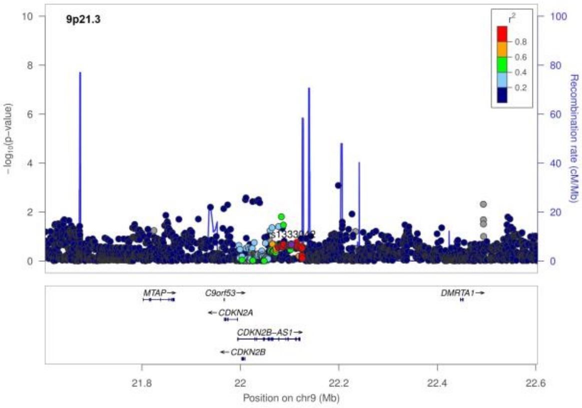
9p21.3
r2
−log10(p−value)
Recombination rate (cM/Mb)
MTAP→
C9orf53→
←CDKN2A
CDKN2B−AS1→
←CDKN2B
DMRTA1→
Position on chr9 (Mb)

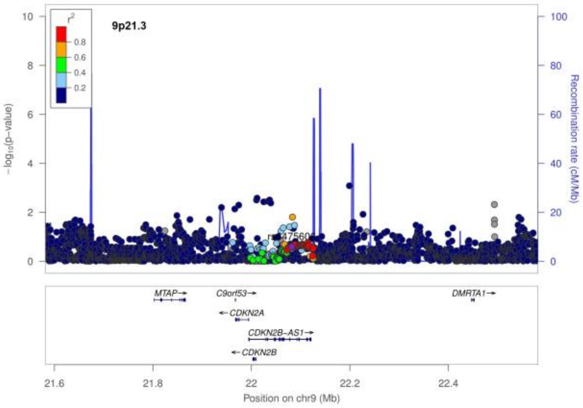
9p21.3
r2
−log10(p−value)
Recombination rate (cM/Mb)
MTAP→
C9orf53→
←CDKN2A
CDKN2B−AS1→
←CDKN2B
DMRTA1→
Position on chr9 (Mb)

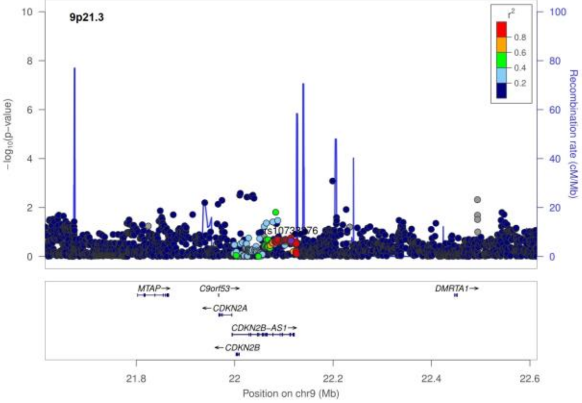
9p21.3
r2
−log10(p−value)
Recombination rate (cM/Mb)
MTAP→
C9orf53→
←CDKN2A
CDKN2B−AS1→
←CDKN2B
DMRTA1→
Position on chr9 (Mb)

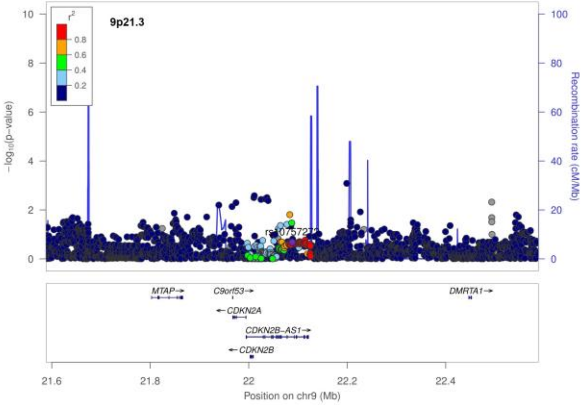
9p21.3
r2
−log10(p−value)
Recombination rate (cM/Mb)
MTAP→
C9orf53→
←CDKN2A
CDKN2B−AS1→
←CDKN2B
DMRTA1→
Position on chr9 (Mb)

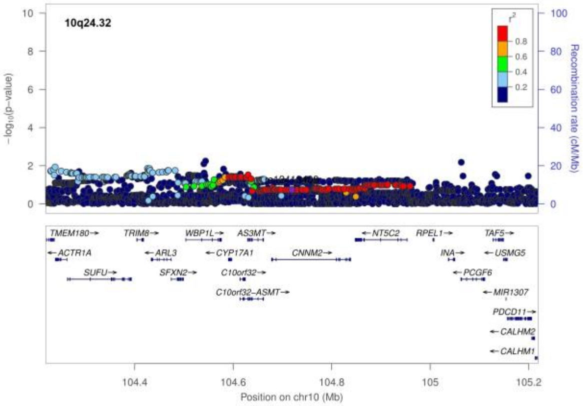
10q24.32
r2
−log10(p−value)
Recombination rate (cM/Mb)
TMEM180→
←ACTR1A
SUFU→
TRIM8→
←ARL3
SFXN2→
WBP1L→
←CYP17A1
AS3MT→
C10orf32→
C10orf32−ASMT→
CNNM2→
←NT5C2
RPEL1→
INA→
←PCGF6
TAF5→
←USMG5
←MIR1307
PDCD11→
←CALHM2
←CALHM1
Position on chr10 (Mb)

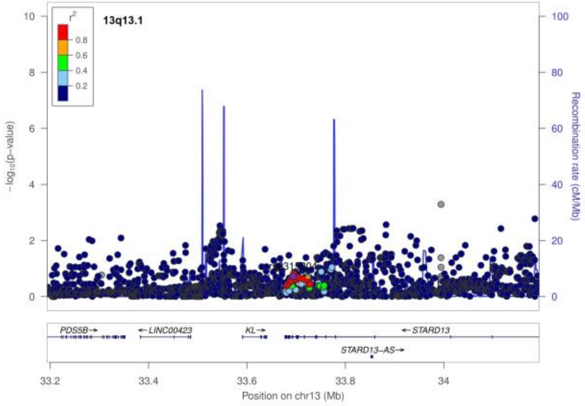
13q13.1
r2
−log10(p−value)
Recombination rate (cM/Mb)
PDS5B→
←LINC00423
KL→
←STARD13
STARD13−AS→
Position on chr13 (Mb)

Supplementary Figure S3. Regional validation of 12 previous GWAS associated loci (23 previous genome-wide significant SNPs)

Supplementary Table S1.

Marker	CHR	POS	Allele1	Allele2	Zscore	P-value	Direction	P.FC	WEIGHT.FC	BETA.FC	SE.FC	P.UI	WEIGHT.UI	BETA.UI	SE.UI	LOCI
**rs76308736**	8	10572792	a	g	-4.753	2.01E-06	--	0.0000045	1945	-1.13246	0.246891	0.0093	4033	0.3	0.12	8p23.1
**rs79244282**	8	10624237	t	c	4.714	2.43E-06	++	0.00000155	1945	-1.28675	0.267805	0.0163	4033	0.3	0.12	8p23.1
**rs7084131**	10	8399121	a	g	4.686	2.78E-06	++	0.00000483	1945	1.03693	0.226803	0.0114	4033	-0.24	0.09	10p14
**rs72753560**	5	31626036	a	g	4.644	3.42E-06	++	0.00000275	1945	0.698537	0.14898	0.0165	4033	-0.15	0.06	5p13.3
**rs4867356**	5	31623146	t	c	-4.608	4.07E-06	--	0.00000491	1945	0.680363	0.148924	0.0148	4033	-0.16	0.06	5p13.3
**rs117537300**	8	10743939	a	g	-4.47	7.81E-06	--	0.000000664	1945	-1.21512	0.244413	0.0466	4033	0.21	0.11	8p23.1
**rs56034913**	6	43619582	a	g	4.326	1.52E-05	++	0.00000238	1945	-1.62618	0.344681	0.0465	4033	0.3	0.15	6p21.1
**rs6940568**	6	43622285	t	c	-4.309	1.64E-05	--	0.00000192	1945	-1.6476	0.346004	0.0525	4033	0.3	0.15	6p21.1
**rs148409800**	6	43621108	t	c	-4.275	1.91E-05	--	0.00000192	1945	-1.6476	0.346004	0.0577	4033	0.29	0.15	6p21.1
**rs114988842**	6	43620110	t	c	-4.23	2.34E-05	--	0.00000192	1945	-1.6476	0.346004	0.0653	4033	0.28	0.15	6p21.1
**rs78339483**	6	43619226	t	c	4.202	2.65E-05	++	0.00000192	1945	-1.6476	0.346004	0.0705	4033	0.28	0.15	6p21.1
**rs6915370**	6	43617995	a	g	4.163	3.14E-05	++	0.00000192	1945	-1.6476	0.346004	0.0781	4033	0.27	0.15	6p21.1
**rs6911069**	6	43617687	a	g	-4.151	3.31E-05	--	0.00000192	1945	-1.6476	0.346004	0.0806	4033	0.27	0.15	6p21.1
**rs8063371**	16	8284980	a	g	4.104	4.07E-05	++	0.00000141	1945	-0.563417	0.116817	0.0997	4033	-0.08	0.05	16p13.2
**rs76079870**	6	43615434	a	g	4.076	4.59E-05	++	0.00000192	1945	-1.6476	0.346004	0.0979	4033	0.26	0.15	6p21.1
**rs142836448**	3	29398714	a	g	4.055	5.01E-05	++	0.00000226	1945	0.786315	0.166286	0.0983	4033	-0.12	0.07	3p24.1
**rs7749866**	6	43605219	a	g	-4.027	5.66E-05	--	0.00000376	1945	-1.53854	0.33271	0.0908	4033	0.26	0.15	6p21.1
**rs7750364**	6	43602008	t	g	-4.022	5.78E-05	--	0.00000376	1945	-1.53854	0.33271	0.092	4033	0.25	0.15	6p21.1
**rs111241794**	6	43602538	t	c	-4.022	5.78E-05	--	0.00000376	1945	-1.53854	0.33271	0.092	4033	0.25	0.15	6p21.1
**rs6938576**	6	43599394	a	g	-4.008	6.13E-05	--	0.00000423	1945	-1.5273	0.332027	0.092	4033	0.25	0.15	6p21.1
**rs962929**	16	8285898	a	t	3.968	7.24E-05	++	0.00000186	1945	-0.558539	0.11713	0.1285	4033	-0.07	0.05	16p13.2
**rs13029421**	2	28823571	t	c	-3.954	7.68E-05	--	0.00000323	1945	-1.35667	0.291423	0.1139	4033	0.18	0.11	2p23.2
**rs142704507**	6	43607074	t	c	-3.95	7.80E-05	--	0.00000286	1945	-1.56469	0.334308	0.119	4033	0.24	0.16	6p21.1
**rs12660473**	6	43611038	a	g	-3.95	7.82E-05	--	0.00000286	1945	-1.56469	0.334308	0.1191	4033	0.24	0.16	6p21.1
**rs3757271**	6	43603468	t	c	-3.949	7.86E-05	--	0.00000286	1945	-1.56469	0.334308	0.1195	4033	0.24	0.16	6p21.1
**rs8044543**	16	8285015	a	t	-3.945	7.99E-05	--	0.00000264	1945	-0.549584	0.117009	0.1234	4033	-0.07	0.05	16p13.2
**rs144339892**	6	43601509	t	c	-3.931	8.46E-05	--	0.00000286	1945	-1.56469	0.334308	0.1247	4033	0.24	0.16	6p21.1
**rs35295534**	2	28829620	a	g	-3.928	8.55E-05	--	0.0000028	1945	-1.34009	0.286047	0.1262	4033	0.17	0.11	2p23.2
**rs11638991**	15	78569950	a	g	3.918	8.93E-05	++	0.00000491	1945	-0.593136	0.129831	0.1102	4033	0.08	0.05	15q25.1
**rs8032417**	15	78569930	a	g	3.865	0.000111	++	0.00000482	1945	-0.593635	0.129826	0.1261	4033	0.08	0.05	15q25.1

**rs1554600**	3	61157774	c	g	-3.852	0.000117	--	4.66E-09	1945	-1.98215	0.338309	0.5345	4033	0.11	0.17	3p14.2
**rs13028204**	2	28853137	c	g	3.846	0.00012	++	0.00000239	1945	-1.38092	0.29275	0.1597	4033	0.16	0.11	2p23.2
**rs982855**	16	8297685	t	c	3.843	0.000122	++	0.00000178	1945	-0.557424	0.116681	0.1735	4033	-0.06	0.05	16p13.2
**rs7190604**	16	8295039	a	g	3.834	0.000126	++	0.00000242	1945	-0.551713	0.11701	0.1635	4033	-0.06	0.05	16p13.2
**rs71443090**	2	28871783	c	g	-3.816	0.000136	--	0.00000441	1945	-1.33072	0.289858	0.1449	4033	0.16	0.11	2p23.2
**rs114999403**	3	131692910	t	g	3.814	0.000137	++	0.00000267	1945	-2.01095	0.428374	0.1666	4033	0.25	0.18	3q22.1
**rs13020458**	2	28857714	t	c	-3.81	0.000139	--	0.00000365	1945	-1.34607	0.290695	0.1548	4033	0.16	0.11	2p23.2
**rs67702090**	2	28866637	a	t	3.805	0.000142	++	0.0000041	1945	-1.33657	0.290173	0.1517	4033	0.16	0.11	2p23.2
**rs11863984**	16	8290916	t	c	3.783	0.000155	++	0.00000211	1945	-0.553815	0.11678	0.1893	4033	-0.06	0.05	16p13.2
**rs116130729**	4	16012786	t	c	-3.778	0.000158	--	0.00000018	1945	-1.79302	0.343542	0.3291	4033	0.2	0.2	4p15.32
**rs77639126**	2	25598423	a	c	3.774	0.000161	++	0.00000235	1945	-1.58283	0.335271	0.188	4033	0.25	0.19	2p23.3
**rs2360850**	16	8293989	a	g	3.754	0.000174	++	0.00000242	1945	-0.551713	0.11701	0.1949	4033	-0.06	0.05	16p13.2
**rs7203131**	16	8296805	a	g	3.747	0.000179	++	0.00000278	1945	-0.54768	0.116865	0.1911	4033	-0.06	0.05	16p13.2
**rs4482268**	16	8297350	t	g	3.743	0.000182	++	0.00000293	1945	-0.546432	0.116866	0.1901	4033	-0.06	0.05	16p13.2
**rs982854**	16	8297724	t	c	3.735	0.000188	++	0.00000285	1945	-0.547261	0.116901	0.1948	4033	-0.06	0.05	16p13.2
**rs2039332**	9	9138642	t	c	-3.735	0.000188	--	0.00000148	1945	-2.05874	0.42766	0.2285	4033	0.41	0.34	9p23
**rs7195828**	16	8295407	a	g	3.726	0.000194	++	0.00000278	1945	-0.54768	0.116865	0.1997	4033	-0.06	0.05	16p13.2
**rs141084492**	3	61158840	t	c	3.715	0.000203	++	1.92E-08	1945	-1.91471	0.340729	0.5346	4033	0.11	0.17	3p14.2
**rs78181937**	2	25605740	c	g	-3.697	0.000218	--	0.00000235	1945	-1.58283	0.335271	0.2214	4033	0.23	0.19	2p23.3
**rs1462099**	16	8298133	a	g	3.656	0.000256	++	0.00000439	1945	-0.53757	0.117072	0.2068	4033	-0.06	0.05	16p13.2
**rs7989887**	13	25681112	t	c	3.613	0.000303	++	0.000000693	1945	-1.00943	0.203382	0.341	4033	0.08	0.08	13q12.13
**rs3002218**	13	25683444	a	g	-3.586	0.000336	--	0.000000731	1945	-1.00686	0.203286	0.3544	4033	0.07	0.08	13q12.13
**rs3002219**	13	25683894	t	c	-3.582	0.000341	--	0.000000731	1945	-1.00686	0.203286	0.3568	4033	0.07	0.08	13q12.13
**rs146929064**	7	44346336	a	c	3.491	0.000481	++	0.00000132	1945	-1.83941	0.38035	0.3725	4033	0.18	0.2	7p13
**rs79515922**	13	25684362	a	g	-3.483	0.000495	--	0.0000017	1945	-0.974484	0.203608	0.359	4033	0.07	0.08	13q12.13
**rs12430973**	13	25687428	a	c	3.477	0.000507	++	0.00000162	1945	-0.977041	0.203704	0.3667	4033	0.07	0.08	13q12.13
**rs61949677**	13	25697959	a	g	-3.45	0.00056	--	0.00000137	1945	-0.985199	0.203994	0.3971	4033	0.07	0.08	13q12.13
**rs9511648**	13	25696076	t	c	-3.446	0.00057	--	0.00000145	1945	-0.982214	0.203877	0.3958	4033	0.07	0.08	13q12.13
**rs9511647**	13	25694482	a	c	-3.444	0.000574	--	0.00000145	1945	-0.982214	0.203877	0.3971	4033	0.07	0.08	13q12.13
**rs2994902**	13	25690566	t	c	3.435	0.000592	++	0.00000172	1945	-0.973997	0.203588	0.3897	4033	0.07	0.08	13q12.13
**rs9511641**	13	25690765	a	t	3.425	0.000616	++	0.00000172	1945	-0.973997	0.203588	0.3969	4033	0.07	0.08	13q12.13
**rs2399431**	3	112280706	c	g	-3.372	0.000747	--	0.000000633	1945	-2.44035	0.489933	0.5182	4033	-0.15	0.23	3q13.2
**rs9511655**	13	25711337	a	g	-3.363	0.000771	--	0.00000271	1945	-0.95984	0.204584	0.4029	4033	0.07	0.08	13q12.13
**rs138031402**	16	8960325	a	g	-3.327	0.000879	--	0.000000887	1945	-2.40373	0.489044	0.5243	4033	0.22	0.35	16p13.2

**rs2705520**	3	112269287	a	g	-3.315	0.000917	--	0.000000633	1945	-2.44035	0.489933	0.564	4033	-0.13	0.23	3q13.2
**rs1877364**	3	112303231	t	c	3.313	0.000924	++	0.000000633	1945	-2.44035	0.489933	0.5659	4033	-0.13	0.23	3q13.2
**rs2638034**	3	112305873	a	g	3.313	0.000924	++	0.000000633	1945	-2.44035	0.489933	0.5659	4033	-0.13	0.23	3q13.2
**rs1385527**	3	112301244	c	g	3.312	0.000925	++	0.000000633	1945	-2.44035	0.489933	0.5661	4033	-0.13	0.23	3q13.2
**rs1472106**	3	112298471	t	c	3.312	0.000926	++	0.000000633	1945	-2.44035	0.489933	0.5664	4033	-0.13	0.23	3q13.2
**rs1532198**	3	112298523	c	g	3.312	0.000927	++	0.000000633	1945	-2.44035	0.489933	0.5665	4033	-0.13	0.23	3q13.2
**rs2969894**	3	112298995	c	g	-3.312	0.000927	--	0.000000633	1945	-2.44035	0.489933	0.5667	4033	-0.13	0.23	3q13.2
**rs2705551**	3	112310253	t	c	-3.311	0.000929	--	0.000000633	1945	-2.44035	0.489933	0.5672	4033	-0.13	0.23	3q13.2
**rs2933139**	3	112294719	t	g	-3.311	0.000931	--	0.000000633	1945	-2.44035	0.489933	0.5675	4033	-0.13	0.23	3q13.2
**rs2705510**	3	112288393	a	g	-3.31	0.000934	--	0.000000633	1945	-2.44035	0.489933	0.5684	4033	-0.13	0.23	3q13.2
**rs2638030**	3	112285190	a	g	-3.309	0.000936	--	0.000000633	1945	-2.44035	0.489933	0.5688	4033	-0.13	0.23	3q13.2
**rs2638038**	3	112295980	a	g	3.309	0.000938	++	0.000000633	1945	-2.44035	0.489933	0.5692	4033	-0.13	0.23	3q13.2
**rs2705506**	3	112291218	t	g	3.308	0.00094	++	0.000000633	1945	-2.44035	0.489933	0.5698	4033	-0.13	0.23	3q13.2
**rs1844864**	3	112311134	t	c	3.308	0.000941	++	0.000000633	1945	-2.44035	0.489933	0.5701	4033	-0.13	0.23	3q13.2
**rs2464612**	3	112284302	t	c	-3.307	0.000944	--	0.000000633	1945	-2.44035	0.489933	0.5707	4033	-0.13	0.23	3q13.2
**rs986883**	3	112317604	a	c	3.303	0.000956	++	0.000000633	1945	-2.44035	0.489933	0.5738	4033	-0.13	0.23	3q13.2
**rs971832**	3	112272647	a	t	3.299	0.000969	++	0.000000633	1945	-2.44035	0.489933	0.5768	4033	-0.13	0.23	3q13.2
**rs2705554**	3	112273546	t	c	3.295	0.000984	++	0.000000633	1945	-2.44035	0.489933	0.5804	4033	-0.12	0.23	3q13.2
**rs1472107**	3	112298345	a	g	-3.294	0.000988	--	0.00000105	1945	-2.36768	0.484976	0.5352	4033	-0.14	0.23	3q13.2
**rs2638025**	3	112269748	a	c	3.294	0.000988	++	0.000000633	1945	-2.44035	0.489933	0.5814	4033	-0.12	0.23	3q13.2
**rs2933140**	3	112250872	a	g	3.293	0.00099	++	0.000000633	1945	-2.44035	0.489933	0.5818	4033	-0.12	0.23	3q13.2
**rs1491629**	3	112258622	t	c	3.293	0.000991	++	0.000000633	1945	-2.44035	0.489933	0.5822	4033	-0.12	0.23	3q13.2
**rs2668216**	3	112295615	a	g	3.293	0.000992	++	0.00000105	1945	-2.36768	0.484976	0.5361	4033	-0.14	0.23	3q13.2
**rs2705514**	3	112321921	t	c	3.29	0.001004	++	0.000000633	1945	-2.44035	0.489933	0.5851	4033	-0.12	0.23	3q13.2
**rs1388442**	3	112257291	a	g	3.289	0.001006	++	0.000000633	1945	-2.44035	0.489933	0.5857	4033	-0.12	0.23	3q13.2
**rs2638041**	3	112248774	t	g	3.289	0.001007	++	0.000000633	1945	-2.44035	0.489933	0.5859	4033	-0.12	0.23	3q13.2
**rs2705563**	3	112258124	a	t	-3.289	0.001007	--	0.000000633	1945	-2.44035	0.489933	0.5859	4033	-0.12	0.23	3q13.2
**rs2638043**	3	112245836	a	g	-3.288	0.001008	--	0.000000633	1945	-2.44035	0.489933	0.5862	4033	-0.12	0.23	3q13.2
**rs2638044**	3	112245295	a	g	-3.266	0.001091	--	0.000000774	1945	-2.4111	0.487894	0.5862	4033	-0.12	0.23	3q13.2
**rs1877362**	3	112331590	a	g	3.234	0.00122	++	0.000000844	1945	-2.39866	0.487047	0.6048	4033	-0.12	0.23	3q13.2
**rs10934177**	3	112332147	t	c	-3.231	0.001232	--	0.000000844	1945	-2.39866	0.487047	0.6072	4033	-0.12	0.23	3q13.2
**rs116704890**	3	112311803	a	g	-3.231	0.001233	--	0.000000633	1945	-2.44035	0.489933	0.6349	4033	0.11	0.23	3q13.2
**rs1552707**	3	112331348	t	c	-3.231	0.001234	--	0.000000844	1945	-2.39866	0.487047	0.6076	4033	-0.12	0.23	3q13.2
**rs114685196**	3	112282665	a	g	3.229	0.001244	++	0.000000633	1945	-2.44035	0.489933	0.637	4033	0.11	0.23	3q13.2

**rs7618978**	3	112330130	t	c	-3.228	0.001249	--	0.000000844	1945	-2.39866	0.487047	0.6105	4033	-0.12	0.23	3q13.2
**rs145651986**	3	112248033	a	g	-3.217	0.001293	--	0.000000633	1945	-2.44035	0.489933	0.6468	4033	0.11	0.23	3q13.2
**rs9511628**	13	25666239	a	g	-3.195	0.001401	--	0.00000275	1945	-1.06966	0.228125	0.5266	4033	0.06	0.1	13q12.13
**rs73098967**	3	61220002	a	g	3.116	0.001835	++	0.000000267	1945	-1.40637	0.273343	0.8258	4033	0.03	0.12	3p14.2
**rs73098971**	3	61223331	a	g	3.112	0.001857	++	0.000000267	1945	-1.40637	0.273343	0.8292	4033	0.03	0.12	3p14.2
**rs1207426**	2	206154365	t	c	3.085	0.002036	++	0.0000041	1945	-0.875028	0.189958	0.5775	4033	-0.04	0.08	2p33.3
**rs11714398**	3	61226363	a	g	-3.077	0.002093	--	0.000000267	1945	-1.40637	0.273343	0.863	4033	0.02	0.12	3p14.2
**rs55994842**	3	61203154	a	g	-3.076	0.002099	--	0.00000108	1945	-1.31068	0.268776	0.7201	4033	0.04	0.12	3p14.2
**rs4688348**	3	61194447	t	c	3.072	0.002128	++	0.00000108	1945	-1.31068	0.268776	0.7239	4033	0.04	0.12	3p14.2
**rs73098963**	3	61218137	a	t	-3.067	0.00216	--	0.000000267	1945	-1.40637	0.273343	0.872	4033	0.02	0.12	3p14.2
**rs73097006**	3	61186195	a	c	-3.066	0.002169	--	0.00000108	1945	-1.31068	0.268776	0.729	4033	0.04	0.12	3p14.2
**rs192887436**	3	61188626	a	g	3.054	0.002256	++	0.00000108	1945	-1.31068	0.268776	0.7398	4033	0.04	0.12	3p14.2
**rs17064638**	3	61186878	a	g	-3.053	0.002267	--	0.00000108	1945	-1.31068	0.268776	0.7412	4033	0.04	0.12	3p14.2
**rs1207415**	2	206169925	t	c	3.031	0.002436	++	0.00000493	1945	-0.86818	0.190067	0.6042	4033	-0.04	0.08	2p33.3
**rs11714472**	3	61226572	a	g	-3.009	0.002617	--	0.000000782	1945	-1.38208	0.279781	0.8154	4033	0.03	0.12	3p14.2
**rs17583011**	13	54424384	a	g	-2.99	0.002792	--	0.00000133	1945	-1.53832	0.318175	0.7778	4033	0.03	0.12	13q14.3
**rs73104225**	3	60995581	t	g	-2.974	0.00294	--	0.00000305	1945	-2.23132	0.478031	0.7044	4033	0.07	0.18	3p14.2
**rs139102048**	3	61196860	t	c	-2.966	0.003013	--	0.00000343	1945	-1.27397	0.274386	0.6987	4033	0.05	0.12	3p14.2
**rs140866572**	3	61197457	t	c	-2.965	0.003025	--	0.00000343	1945	-1.27397	0.274386	0.6998	4033	0.05	0.12	3p14.2
**rs56174846**	3	61225234	t	c	-2.956	0.003117	--	0.000000782	1945	-1.38208	0.279781	0.8663	4033	0.02	0.12	3p14.2
**rs73098993**	3	61230478	a	g	2.94	0.003283	++	0.00000141	1945	-1.33911	0.277653	0.8185	4033	0.03	0.12	3p14.2
**rs73098988**	3	61227542	t	c	-2.923	0.003467	--	0.000000782	1945	-1.38208	0.279781	0.898	4033	0.02	0.12	3p14.2
**rs116375594**	3	61197014	c	g	2.902	0.003705	++	0.00000343	1945	-1.27397	0.274386	0.7574	4033	0.04	0.12	3p14.2
**rs114375292**	13	54413853	t	c	2.847	0.004414	++	0.000000725	1945	-1.55348	0.313554	0.9797	4033	0	0.13	13q14.3
**rs1357639**	3	61211013	a	t	2.794	0.005211	++	0.0000018	1945	-1.25222	0.262258	0.9319	4033	0.01	0.12	3p14.2
**rs73161791**	22	36455779	t	c	-2.705	0.006829	--	0.00000365	1945	-2.59289	0.559998	0.938	4033	0.02	0.29	22q12.3
**rs11699714**	20	1613011	t	c	2.647	0.008133	++	0.00000367	1945	-1.18146	0.255218	0.9942	4033	0	0.11	20p13
**rs55952004**	20	1614600	a	t	-2.625	0.008671	--	0.000004716	1945	-1.16432	0.254383	0.9864	4033	0	0.11	20p13
**rs56040592**	20	1611918	a	g	-2.591	0.009575	-+	0.00000198	1945	-1.22327	0.257248	0.8822	4033	-0.02	0.11	20p13
**rs2705535**	3	112208927	t	c	-2.59	0.009588	-+	0.00000327	1945	-1.86427	0.40064	0.9381	4033	-0.01	0.18	3q13.2
**rs10495046**	1	217417902	a	g	-2.563	0.01037	-+	0.00000474	1945	-1.18702	0.259403	0.9545	4033	-0.01	0.11	1q41
**rs12058987**	1	217421015	a	c	-2.107	0.03515	-+	0.00000133	1945	-1.47758	0.305576	0.4277	4033	-0.1	0.13	1q41
**rs79571254**	8	127902373	t	c	-1.899	0.05762	-+	0.00000162	1945	0.706359	0.147273	0.3082	4033	0.06	0.06	8q24.21
**rs6772523**	3	61190420	a	g	1.867	0.06185	+-	0.00000492	1945	-0.949471	0.207846	0.3687	4033	-0.09	0.1	3p14.2

**rs59910027**	3	61194299	t	g	1.857	0.06336	+-	0.00000492	1945	-0.949471	0.207846	0.3618	4033	-0.09	0.1	3p14.2
**rs112000237**	8	127911357	a	g	1.854	0.06378	+-	0.00000162	1945	0.706359	0.147273	0.283	4033	0.07	0.06	8q24.21
**rs150148362**	1	234191488	a	g	-1.831	0.06716	-+	0.000000816	1945	-2.55442	0.517978	0.2317	4033	-0.41	0.35	1q42.2
**rs56268052**	3	61215230	a	g	-1.78	0.07513	-+	0.00000283	1945	-0.978093	0.208875	0.2778	4033	-0.1	0.1	3p14.2
**rs7011138**	8	127922200	a	t	-1.579	0.1143	-+	0.00000135	1945	0.72319	0.149667	0.1518	4033	0.09	0.06	8q24.21
**rs151319278**	13	44537888	a	g	-1.11	0.2671	-+	0.00000458	1945	-1.89937	0.414408	0.067	4033	-0.45	0.25	13q14.3

Supplementary Table S2. FBAT-O result of 50 *FHIT* variants with significant association ($p<0.05$) in Nunavik Inuit

**Marker**	**chr**	**pos**	**afreq**	**fam#**	**S-E(S)**	**Var(S)**	**Z**	**P**	**Offset**
rs780365	3	59761789	0.377	14	6.842	5.254	2.985	0.002839	0.231
rs17361653	3	59771515	0.462	18	-6.903	5.489	-2.946	0.003216	0.203
rs9809480	3	60448671	0.23	14	5.032	3.155	2.833	0.004615	0.277
rs1597104	3	60453227	0.23	14	5.032	3.155	2.833	0.004615	0.277
rs7621114	3	60418721	0.205	13	4.663	3.003	2.691	0.007123	0.257
rs2255772	3	60557852	0.207	10	4.039	2.519	2.545	0.010928	0.245
rs2687189	3	60423009	0.438	18	4.701	3.768	2.422	0.015436	0.095
rs445057	3	60447369	0.433	18	4.701	3.768	2.422	0.015436	0.095
rs424675	3	60448261	0.433	18	4.701	3.768	2.422	0.015436	0.095
rs17061176	3	59755841	0.179	11	4.311	3.188	2.414	0.015763	0.224
rs13092167	3	59772342	0.293	10	4.244	3.148	2.392	0.016762	0.212
rs3920477	3	60733152	0.328	15	4.425	3.484	2.371	0.017762	0.154
rs9861525	3	60004908	0.398	15	4.756	4.157	2.332	0.019677	0.268
rs241700	3	60516256	0.311	13	4.01	3.005	2.313	0.020728	0.235
rs10451993	3	60566492	0.321	13	4.01	3.005	2.313	0.020728	0.235
rs17669257	3	60566161	0.412	17	4.626	4.158	2.269	0.023295	0.271
rs17609699	3	60487578	0.48	18	-5.16	5.188	-2.265	0.023496	0.27
rs49413	3	59757484	0.418	20	5.311	5.546	2.255	0.024112	0.264
rs993000	3	59745585	0.158	10	2.989	1.815	2.218	0.026535	0.4
rs1683346	3	59749703	0.158	10	2.989	1.815	2.218	0.026535	0.4
rs17061173	3	59754178	0.156	10	2.989	1.815	2.218	0.026535	0.4
rs994931	3	59741758	0.319	15	-4.661	4.506	-2.196	0.028127	0.451
rs780366	3	59762116	0.347	15	-4.661	4.506	-2.196	0.028127	0.451
rs11130733	3	59767786	0.347	15	-4.661	4.506	-2.196	0.028127	0.451
rs17361780	3	59772328	0.347	15	-4.661	4.506	-2.196	0.028127	0.451
rs1627302	3	60658608	0.473	17	-4.48	4.468	-2.119	0.034058	0.27
rs721207	3	60607896	0.468	16	-5.031	5.672	-2.112	0.034649	0.372
rs780354	3	59772460	0.366	15	-4.505	4.578	-2.106	0.035223	0.478
rs2682931	3	60631936	0.359	19	-4.939	5.68	-2.072	0.038225	0.261
rs6766789	3	60090013	0.468	20	-4.352	4.463	-2.06	0.039397	0.349
rs10780042	3	60090402	0.468	20	-4.352	4.463	-2.06	0.039397	0.349
rs9881736	3	60487247	0.139	11	3.056	2.21	2.055	0.039836	0.5
rs6786100	3	60351746	0.402	17	4.528	5.083	2.008	0.04461	0.302
rs2142301	3	60095070	0.396	19	-3.978	4.074	-1.971	0.048745	0.18
rs2885865	3	60610341	0.381	19	-4.741	5.811	-1.967	0.049201	0.292

rs2363670	3	60611613	0.372	19	-4.741	5.811	-1.967	0.049201	0.292
rs7633853	3	60613659	0.38	19	-4.741	5.811	-1.967	0.049201	0.292
rs11926787	3	60615310	0.368	19	-4.741	5.811	-1.967	0.049201	0.292
rs6762641	3	60616153	0.356	19	-4.741	5.811	-1.967	0.049201	0.292
rs10510852	3	60616790	0.356	19	-4.741	5.811	-1.967	0.049201	0.292
rs12637393	3	60620284	0.368	19	-4.741	5.811	-1.967	0.049201	0.292
rs2856058	3	60625598	0.36	19	-4.741	5.811	-1.967	0.049201	0.292
rs1735448	3	60647540	0.36	19	-4.741	5.811	-1.967	0.049201	0.292
rs1716709	3	60657213	0.349	19	-4.741	5.811	-1.967	0.049201	0.292
rs1716714	3	60660179	0.349	19	-4.741	5.811	-1.967	0.049201	0.292
rs1735443	3	60662282	0.349	19	-4.741	5.811	-1.967	0.049201	0.292
rs1735444	3	60664075	0.348	19	-4.741	5.811	-1.967	0.049201	0.292
rs1716722	3	60681361	0.349	19	-4.741	5.811	-1.967	0.049201	0.292
rs1735457	3	60683737	0.349	19	-4.741	5.811	-1.967	0.049201	0.292
rs1735468	3	60703616	0.36	19	-4.741	5.811	-1.967	0.049201	0.292

Supplementary Table S3. Mutation burden of exonic variants of the 4 genes from 18q11.2 loci in FC IA compared with controls

**Loci**	**Gene**	**Number of exonic variants**	**MAC in each gene**	***P*-value**	**std error (SE)**	**permutations**
**18q11.2**	*GATA6*	2	2	0.731269	0.441789	1000
**18q11.2**	*CTAGE1*	6	174	0.0753849	0.371797	5000
**18q11.2**	*RBBP8*	6	43	0.0585883	0.376427	5000
**18q11.2**	***CABLES1***	3	68	**0.00559888**	0.356104	5000

Supplementary Table S4. *CCDC80* exonic and splicing variants in FC IA cases and controls

**Position**	**Annotation**	**MAF (CEU)**	**SNP**	**MAC in case**	**MAC in control**
3:112324305	c.T2812C:p.Y938H	0.01	rs114697626	0	4
3:112328856	c.C2394G:p.L798L	0.002	rs61732242	0	2
3:112357336	c.C1417T:p.H473Y	0.009	rs116307644	4	1
3:112357537	c.A1216G:p.R406G	0		0	1
3:112357730	c.G1023T:p.L341F	0.01	rs115738438	5	2
3:112357878	c.C875A:p.A292E	0		0	1
3:112358168	c.G585C:p.E195D	0	rs370290734	0	1

3:112358171	c.T582A:p.G194G	0.01	rs116819563	5	2
3:112358658	c.G95A:p.S32N	0.001	rs75074453	0	1
3:112358761	UTR5 c.-9G>T	0		0	1
